# A functional bimodal mesoporous silica nanoparticle with redox/cellulase dual-responsive gatekeepers for controlled release of fungicide

**DOI:** 10.1038/s41598-023-27396-8

**Published:** 2023-01-16

**Authors:** Weilan Huang, Hua Pan, Zhongxuan Hu, Meijing Wang, Litao Wu, Fang Zhang

**Affiliations:** Faculty of Environment and Life, Beijing University of Technology, Beijing, 100124 China

**Keywords:** Environmental sciences, Nanoscience and technology

## Abstract

Integrating toxic fungicide into a functional stimuli-responsive nanosystem can effectively improve the fungus control specificity and reduce the effect on non-target organisms. We report here a redox and cellulase dual-responsive multifunctional nanoparticle based on bimodal mesoporous silica (BMMs) to deliver prochloraz (Pro) for the smart management of wilt disease (Pro-AC-SS-BMMs, known as P-ASB). The surface of the nanocarrier was modified with an aminosilane coupling agent, and Pro was encapsulated by physical adsorption using 2,2′-dithiodiacetic acid as a smart bridge and disulfide (SS) cross-linked aminocellulose (AC) as gatekeepers. P-ASB nanoparticles (NPs) had a spherical structure, and the size was 531.2 ± 4.9 nm. The loading rate of Pro was 28.5%, and the NPs possessed excellent redox/cellulase dual-responsive release characteristics in the presence of glutathione (GSH) and cellulase. The nanocarrier could effectively protect Pro against photodegradation and had better foliar wettability than the Pro technical. Fluorescence tracer results showed that the nanocarriers were taken up and activated by the mycelium. P-ASB NPs had better control efficacy against *Rhizoctonia solani* and had no significant toxicity to cells and bacteria. This study provides a new strategy for enhancing the environmental protection and promoting the development of green agriculture.

## Introduction

Pesticides play an indispensable role in the management of crop diseases and increasing crop yield in modern agriculture. However, the utilization efficiency of synthetic pesticides is low, and a large amount of pesticide is lost in field applications owing to poor water dispersibility and instability^1–3^. In addition, long-term inefficient application of pesticides can cause tolerance in target organisms and harm the environment, leading to food security issues^4–6^. Therefore, it is crucial to accurately deliver the pesticide to the target part of the plant and increase its effectiveness in the field^1^.

The rapid development of nanotechnology in materials fields provides an innovative dimension to solve these problems. Mesoporous silica-based nanoparticles are an ideal carrier candidate and have been widely used in diversified fields to deliver cargos due to their excellent textural properties and structural parameters^1^. Bimodal mesoporous silica (BMMs) is mesoporous silica material with dual channel structure^7^. They have 3-nm worm-like holes and 10–30-nm spherical particle accumulation holes. BMMs can be absorbed and conducted by plants due to the nanoparticle size ranging between 20 and 50 nm, which is an obvious feature compared with traditional mesoporous silica materials.

Recently, a range of environmental-stimulus-responsive nanocomposites with intelligently slow/controlled-release were deigned to significantly improve the effective utilization of pesticides^8,9^. The gatekeeper acts as a blocking component at the entrance of the pores of the nanoparticles. Stimuli-responsive systems for “on-demand” release cargos respond to trigger factors such as pH^10,11^, temperature, UV light^12^, redox^13^, and magnetic and electric mediators^14,15^. Glutathione (GSH) is a key antioxidant that extensively distributes in various organisms due to its high antioxidant properties^16,17^. The disulfide bond (–S–S–) is destroyed by the reductive hydrogen in the sulfhydryl group (–SH–) in GSH, and then reduced to the sulfhydryl group^18,19^. Amino cellulose is derived from cellulose and can also be degraded by the cellulase secreted by plant pathogens^20,21^. Its abundant amino functional groups endow it with more physical and chemical properties such as connecting fluorescent groups, good water solubility, and film-forming properties^22,23^. Combining the microenvironment surrounding the fungus with the sustained-release nanotechnology is an available way to control pathogenic fungi, which can release loaded pesticides to specific targets by simulating fungal microenvironment^24–26^.

*Rhizoctonia solani* (*R. solani*) is one of the most destructive soil-borne plant pathogens, which can parasitize 263 species of plants and cause various diseases, such as brown spot, sheath blight and root rot^11,27^. It seriously affects the growth of many commercial crops in agricultural production^11^. Rice sheath blight caused by *R. solani* reduced rice yield by 10–30%, especially in South China and the Yangtze River basin, where that was reduced by up to 50%. The general incidence rate of maize sheath blight was 70–100%, resulting in a yield loss of 10–20%^28^. Prochloraz (Pro), a broad-spectrum imidazole fungicide, effectively prevents and defends plant diseases caused by fungi such as wilt disease. However, Pro has several disadvantages: it is insoluble in water, easily degraded, and poorly absorbed and transported by plants^29,30^. This leads to a short validity period and low utilization rate. The use of smart delivery nanosystems based on mesoporous silica materials as stimuli-responsive factors to trigger the release of fungicide is an important strategy to improve the utilization of Pro and effectively control crop diseases^31,32^.

**Figure 1:**
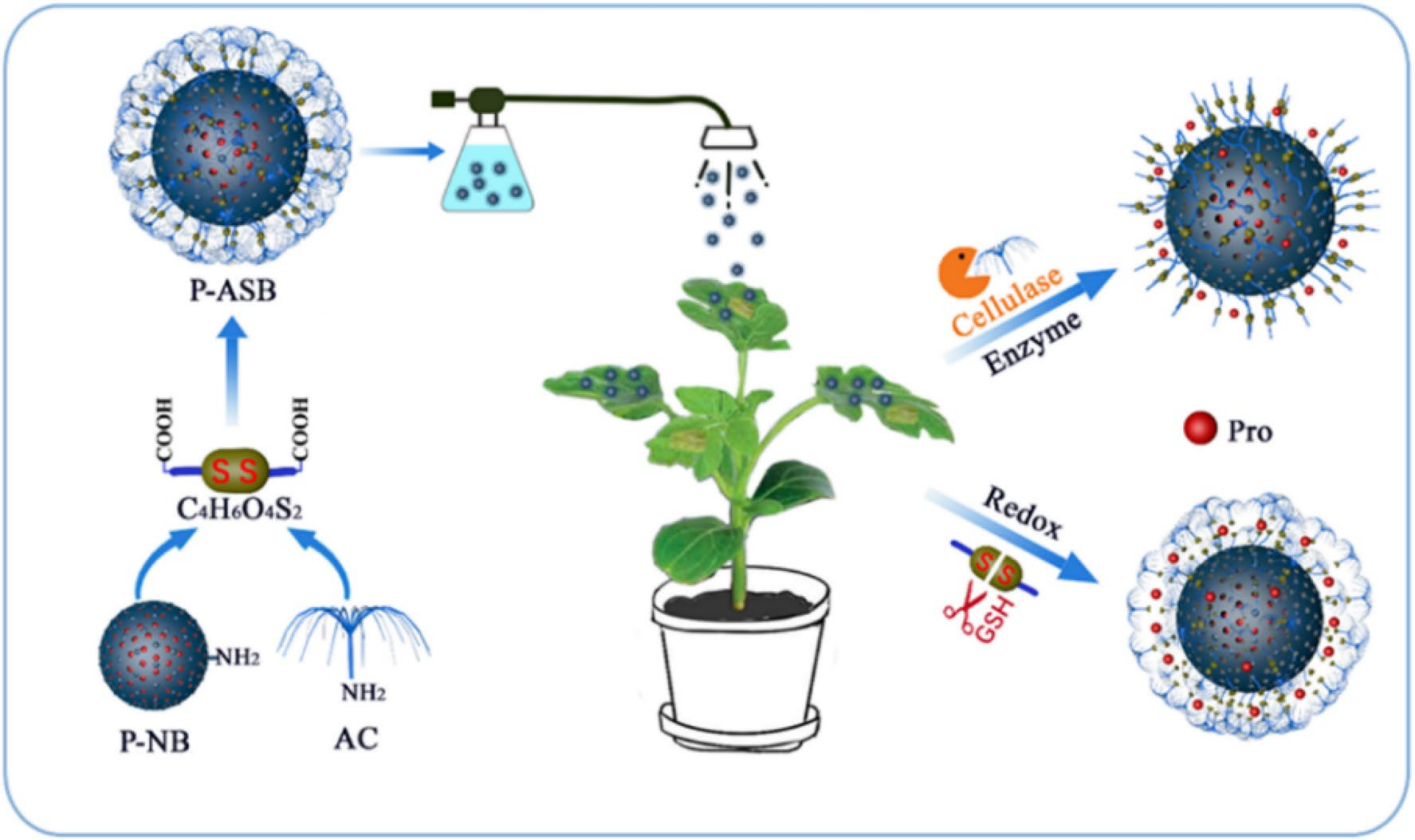
Schematic diagram for preparation of P-ASB nanoparticle and redox/cellulase dual-responsive release mechanism.

Herein, a pathogen microenvironment stimuli-responsive nanosystem was constructed based on BMMs and disulfide cross-linked amino cellulose as gatekeepers was fabricated to deliver Pro for *R. solani* management (Fig. 1). The resulting P-ASB NPs were fully characterized, and the effects of GSH, pH, and cellulase on release profiles as well as the UV-shielding properties, leaf adhesion, uptake, and translocation by the fungi were investigated in detail. Moreover, the targetability, prevention, and control efficacy of the obtained P-ASB nanoparticles against tomato sheath blight, as well as their toxicity to *Escherichia coli* (*E. coli*) and human bronchial epithelial (16HBE) cells, were investigated via carefully designed in vitro and in vivo experiments. This research proved that P-ASB has potential application prospects in the intelligent control of crop diseases.

## Materials and methods

### Materials

Cellulose (98%) and 2,2′-Disulfanediyldiacetic acid were obtained from Shanghai Bide Medical Technology Co., Ltd. (Shanghai, China). Fluorescein isothiocyanate (FITC, 90%), 3-Aminopropyltriethoxysilane (APTES, 98%) and ethylenediamine diacetate (98%) were purchased from Beijing Honghu United Chemical Products Co., LTD (Beijing, China). Tetraethyl orthosilicate (TEOS, 99.9%), cetyl trimethyl ammonium bromide (CTAB, 98%), and other organic solvents were obtained from Beijing J&K Scientific Technology Co., Ltd. (Beijing, China). Cellulase (50 µg/mg) and glutathione (GSH, 99%) were received from Beijing Minida Technology Co., LTD (Beijing, China). N-(3-dimethylaminopropyl)-N′-ethylcarbodiimide hydrochloride (EDC, 98%) and N-hydroxysuccinimide (NHS, 98%) and were purchased from Aladdin Co. Ltd (Shanghai, China). Prochloraz technical (Pro TC, 97.5%) was purchased from Beijing Jinyue Biotechnology Co., Ltd (Beijing, China). *Rhizoctonia solani* (strain number, 3.2871) was purchased from China General Microbiological Culture Collection Center, Chinese Academy of Sciences.

### Synthesis of nanoparticles

#### Synthesis of NH_2_-BMMs (NB)

The BMMs was synthesized according to a previous report^10^. To graft amine groups onto the BMMs, BMMs (1.0 g) were dissolved in 100 mL of methylbenzene, and 500 µL of APTES was added dropwise. The mixture was stirred at 70 °C for 4 h. Then, the products was washed with methanol to remove free APTES and the product (NH_2_-BMMs, named as NB) was collected after drying at 50 °C under vacuum for 12 h.

#### Synthesis of Pro@NH_2_-BMMs (P-NB) NPs

Pro (0.4 g) was dissolved in hexane (20 mL), and the synthesized NB (0.2 g) was dispersed in the hexane solution with stirring for 24 h. Subsequently, the mixture was filtered and washed with warm hexane to remove Pro adsorbed on the surface of nanoparticles. The resulting Pro@NH_2_-BMMs (P-NB) was centrifuged at 10,000 rpm for 10 min and dried at 50 °C for 5 h.

#### Synthesis of aminocellulose (AC)

Cellulose (2.02 g) was dispersed in 47 mL of dimethyl acetamide with nitrogen protection, and stirred at 160 °C for 1 h until the temperature dropped to 100 °C. Subsequently, 4.0 g of LiCl was dispersed in the mixture with constant stirring to room temperature. After the cellulose was completely dissolved, 15.0 g of triethylamine (Et3N) and 4.04 g of 4-toluene sulfonyl chloride (TsCl) were added to the solution, and the reaction was conducted for 24 h at 8 °C. The reaction solution was then poured into ice water to precipitate, and the precipitate is filtered, washed and dried. Next, 3.1 g of precipitate was dissolved in 30 mL of dimethyl sulfoxide and the reaction was carried out for 6 h at 100 °C after adding 30.0 g of ethylenediamine. The reaction solution was then poured into acetone, and the crude product was filtered and washed several times with isopropanol. The collected products were dried in vacuum to obtain aminocellulose (AC).

#### Synthesis of Pro@AC-SS-BMMs (P-ASB) NPs

First, 0.078 g of 2,2′-Disulfanediyldiacetic acid, 0.35 g of EDC and 0.23 g of NHS were dissolved in 62 mL of deionized water and stirred for 30 min. Next, 0.2 g of P-NB was mixed and stirred medially for 24 h at room temperature. The resulting dispersions were centrifuged and washed with deionized water multiple times. After the intermediate was dispersed in 20 mL of deionized water, 0.23 g of NHS and 0.35 g of EDC were dispersed again in the dispersions and stirred in ice bath for 30 min. Then, 10 mL of AC solution (20 mg/mL) was dissolved and stirred for 24 h at 25 °C. The final dispersions were centrifuged and washed with deionized water multiple times. The yellowish residue Pro@AC-SS-BMMs (P-ASB) was dried at 50 °C for 12 h. When Pro was not loaded in P-ASB, the obtained product was used as a blank control.

#### Synthesis of fluorescently labeled AC-SS-BMMs/FITC (ASB/FITC)

The nanocarriers were easily functionalized with FITC via the amine group (–NH_2_) on the surface of ASB. ASB (200 mg) and FITC (50 mg) were suspended in 25 mL of ethanol, and then, the mixed solution was stirred for 24 h at 25 °C in the dark. FITC-labeled nanoparticles (designated as AC-SS-BMMs/FITC) were centrifuged at 7000 rpm for 10 min and washed with ethanol. The obtained ASB/FITC NPs were then stored away from light at room temperature.

#### Characterizations of P-ASB NPs

The morphology and structure of the nanoparticles were taken by a transmission electron microscopy (TEM, JEOL, Tokyo, Japan) with a 200 kV accelerating voltage. An Autosorb-iQ pore analyzer was used to detect nitrogen (N_2_) adsorption–desorption isotherms (Quantachrome, Boynton Beach, FL, USA). The specific surface areas and pore-size distribution of Nps were analyzed separately using the Brunauer–Emmett–Teller (BET) method and the Barrett-Joyner-Halenda (BJH) method. The characteristic basal reflection patterns of NPs were carried out using an D8 ADVANCE X-ray powder diffractometer (XRPD, Karlsruhe, Germany) with Ni-filtered Cu *K*α radiation. A Nicolet Nexus 470 Fourier Transform Infrared Spectroscopy (FTIR) spectrometer was used to assay the chemical functional groups of the samples (Nicolet Instrument Corp., Concord, CA, USA). The elemental compositions and contents in the nanoparticles were analyzed by Raman spectroscopy (Wasatchphotonics, Logan, UT, USA) and X-ray photoelectron spectroscopy (XPS) (Kratos Ltd., Manchester, UK) was achieved by a photoelectron spectrometer (ESCALab 250Xi, Thermo Fisher Scientific, Waltham, USA). The Zeta potential and hydrate particle sizes of nanoparticles were detected using dynamic light scattering (DLS) by a Zeta Sizer Nano ZS analyzer (Malvern Instruments Ltd., Malvern, UK). Thermo gravimetric analysis (TGA) was performed by a thermal analyzer (PerkinElmer, Waltham, MA, USA) from 30 to 800 °C with a heating rate of 10 °C min^−1^ under nitrogen atmosphere.

#### Pro loading content

For Pro loading, 20 mg of P-ASB was added in 60 mL of methanol and ultrasonicated for 3 h. Subsequently, the mixed solution was centrifugated at 12,000 rpm for 10 min and the supernatant was obtained. A high performance liquid chromatography (HPLC, Agilent Corp., Santa Clara, CA, USA) was used to determine the concentration of Pro. The chromatographic separation was assayed at 220 nm using a C18 reversed-phase column (5 µm × 4.6 mm × 250 mm), and the mobile phase consisted of acetonitrile/0.1% acetic acid (70:30, v/v) at a flow rate of 1 mL min^−1^.

#### Release behavior

The release performances of Pro from P-ASB nanosystem were investigated using a dialysis method. Firstly, the effects of different pH values (5.0, 7.0 and 9.0) on the release of nanosystem were investigated. Then, the release buffer solutions with different GSH concentrations (2.5 mM and 5.0 mM), as well as cellulase (0.5 mg/mL) and cellulase (0.5 mg/mL) with GSH (5 mM), were prepared at pH 7.0. Seven groups of 10 mg of P-ASB were weighed and dispersed with 4 mL of different release media and transferred to dialysis bags. The sealed dialysis bags were immersed in the same solution (46 mL) as their interior. The entire system was stirred at 100 rpm at room temperature to release Pro. Then, 1 mL of supernatant was periodically removed for Pro solubility determination, and the new equivalent release medium was supplied immediately after sampling to make sure that the release volume was always maintained at 50 mL. The experiment was repeated three times for each group.

#### Photostability

The photolytic properties of P-ASB NPs were tested with Pro as the control. Ten groups of the same quality samples were added in 25 mL of phosphate buffer solution (PBS) with 0.1% tween-80 and transferred to a quartz tube for sealing. These mixtures were exposed to a 36 W, 254 nm germicidal lamp at room temperature. Dispersions were periodically removed, and ultrasonicated for 3 h. Subsequently, the solution was centrifugated at 12,000 rpm for 10 min, and the supernatant was measured by HPLC.

#### Uptake of NPs in fungi

FITC was used to track ASB for fluorescence labeling to verify whether the nanocarriers can be taken up by *R. solani*. Briefly, 5-mm-diameter mycelial disc cakes of *R. solani* were inoculated in potato dextrose agar (PDA) petri dishes with 5 (mg/mL) ASB/FITC NPs at 25 °C with 60% humidity. The fungi were observed through a TCS-SP8 confocal laser scanning microscope (CLSM, Leica, Wetzlar, Germany) at an excitation wavelength of 488 nm for 7 days. The mycelium cultured with deionized water was used as a negative control.

#### Dynamic contact angle analysis

To further study the adhesion property of P-ASB NPs on the leaf surface, the dynamic contact angle of the NPs was determined by a contact-angle meter (Dataphysics-TP50, Dataphysics Ltd, Stuttgart, Germany). Here, 5 mg of NPs was added in 10 mL deionized water, and 4 µL of P-ASB NPs suspension was dropped onto tomato leaves. The images of the drops on the leaves within 1 min were taken quickly, and the dynamic contact angles were recorded. Pro TC was used as a positive control, and BMMs and deionized water were used as negative controls.

#### Fungicidal activity

The antibiological activity of P-ASB NPs against *R. solani* was tested via a growth-inhibition assay. A 5 mm-diameter mycelial discs were seeded separately at the center of PDA plates with different concentrations (1.0, 0.5, 0.25, 0.125 and 0.0625 mg/L) of P-ASB NPs. Pro TC and deionized water served as controls. After incubating at 25 °C for 12 days, the diameter of colonies was measured by cross-crossing method, and the relative inhibition rate of P-ASB NPs was calculated according to the colony diameter^33^. The IC50 values were obtained by linear regression equation.

#### Biosafety evaluation

To further evaluate the biosafety properties of ASB NPs, human bronchial epithelial (16HBE) cells were incubated in 1640 media containing 10% fetal bovine serum (FBS) at 37 °C for 24 h. The cells were disposed with ASB NPs at various concentrations (500, 250, 125, 62.5, 31.25 and 0 mg/L for 24 h. A cell counting kit (CCK8, Beijing Solarbio Technology Co., Ltd, Beijing, China) was used to determine the cell viability. *Escherichia coli* (*E. coli*) was cultured at 37 °C for 24 h in Luria–Bertani (LB) culture media with different concentrations of ASB NPs as described above. A UV–Vis absorptiometry (Eppendorf Biophotometer plus, Hamburg, Germany) was utilized to detect the optical density (OD) values of *E. coli*, and the detection wavelength was at 600 nm. Deionized water served as a blank control. Biological activities were evaluated by the following equation: Relative viability (%) = (absorbance value of sample − absorbance value of medium)/(absorbance value of control − absorbance value of medium) × 100%.

## Results and discussion

### Preparation and characterization

**Figure 2:**
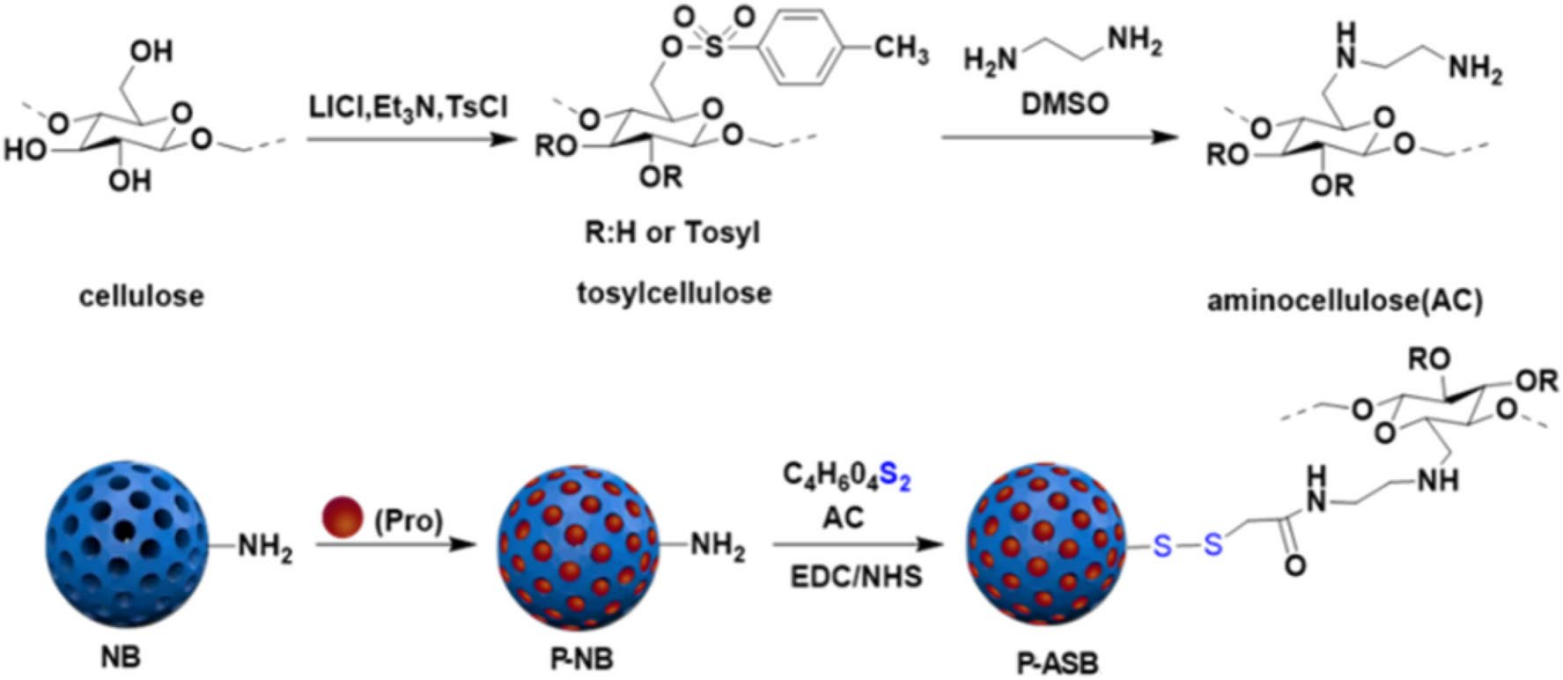
The mechanism for preparation of the P-ASB NPs.

The preparation process of nanoparticles is illustrated in Fig. 2. The water-soluble amino cellulose was first synthesized by two substitution reactions of p-toluenesulfonyl group and ethylenediamine on cellulose. Then, ethyl orthosilicate was used as a silicon source, and CTAB was used as the pore-forming agent to produce BMMs with dual-mode mesoporous silicon under alkaline conditions. Amino functional groups were introduced on the surface of BMMs after removal of CTAB at high temperature. Pro was loaded into NB channels via the impregnation adsorption method. Disulfide bonds and AC were first grafted onto P-NB nanoparticles by two amide coupling reactions, and redox and cellulase dual stimuli-responsive P-ASB NPs were obtained.

**Figure 3:**
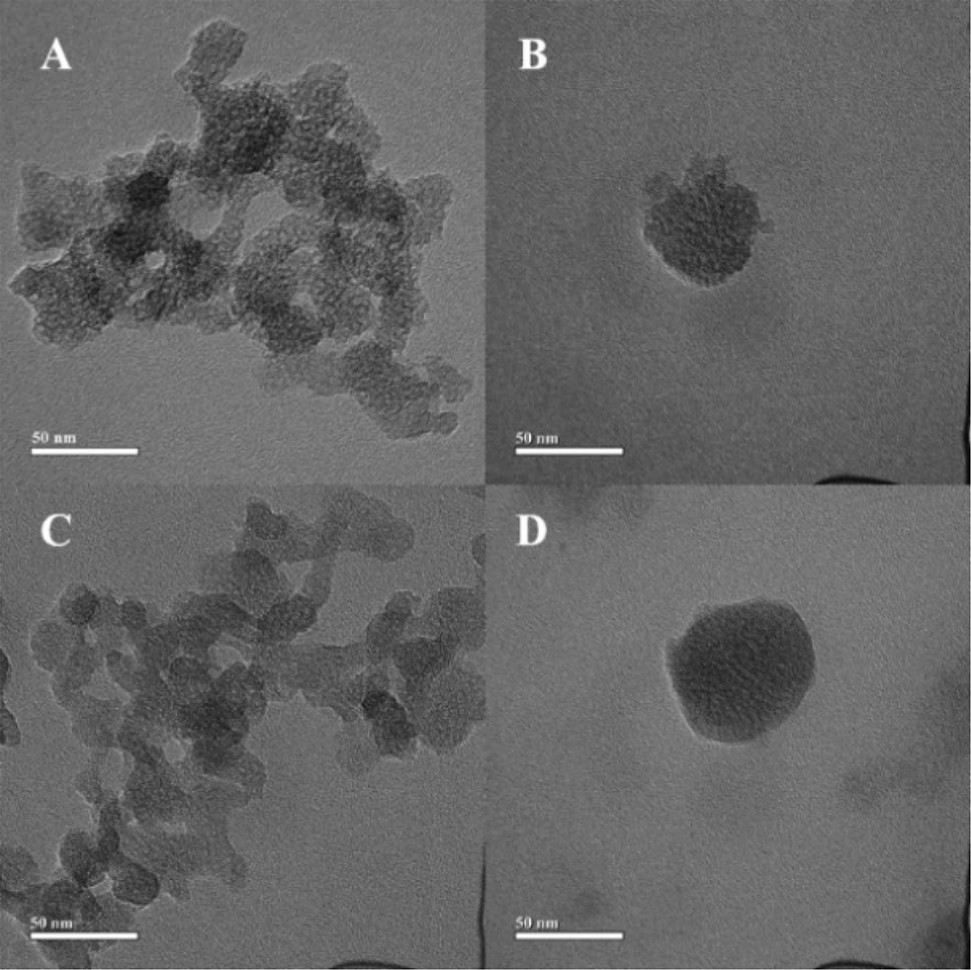
TEM images of BMMs (**A**, **B**) and P-ASB NPs (**C**, **D**).

Transmission electron microscopy (TEM) images of BMMs and P-ASB NPs shown in Fig. 3 indicated that the morphology of NPs was relatively regular with a mean size of 20–50 nm. The pores could be clearly observed in BMMs, but it disappeared after.

Pro was successfully loaded in the BMMS. Compared with BMMs with regular spherical shapes, there was no significant difference in P-ASB morphology, indicating that the introduction of gatekeepers and cargo did not damage the structure and morphology of BMMs. As shown in Table 1, the average hydrodynamic sizes of BMMs and P-ASB NPs in water were 255.0 ± 5.3 nm and 531.2 ± 4.9 nm, respectively. The average hydrodynamic size of P-ASB was larger than that of BMMs because the outer polymer was swollen in water. The zeta potential of BMM nanocomponent was − 24.4 mV due to the deprotonation of Si–OH, which was reversed to + 3.1 mV after APTES modification. After loading of Pro, the zeta potential slightly increased to + 4.2 mV due to the positive zeta potential of Pro. Following the coatings of gatekeepers, the zeta potential of P-ASB increased to + 13.0 mV, which may be the result of –NHC_2_H_4_NH_2_ protonation.

**Table 1:** Hydrodynamic sizes, zeta potentials and characteristic parameters of mesoporous structure of nanoparticles.

Samples	Size (nm)	Zeta potential (mV)	S_BET_ (m^2^/g)	V_t_ (cm^3^/g)	Small pore (nm)	Large pore (nm)
BMMs	255.0 ± 5.3	− 24.40 ± 0.47	1013.76	1.92	2.72	21.03
NB	342.0 ± 3.7	3.06 ± 0.42	566.10	1.18	2.08	18.82
P-NB	458.7 ± 5.1	4.23 ± 0.29	172.42	0.69	–	18.67
P-ASB	531.2 ± 4.9	13.0 ± 0.36	132.94	0.56	–	17.80

**Figure 4:**
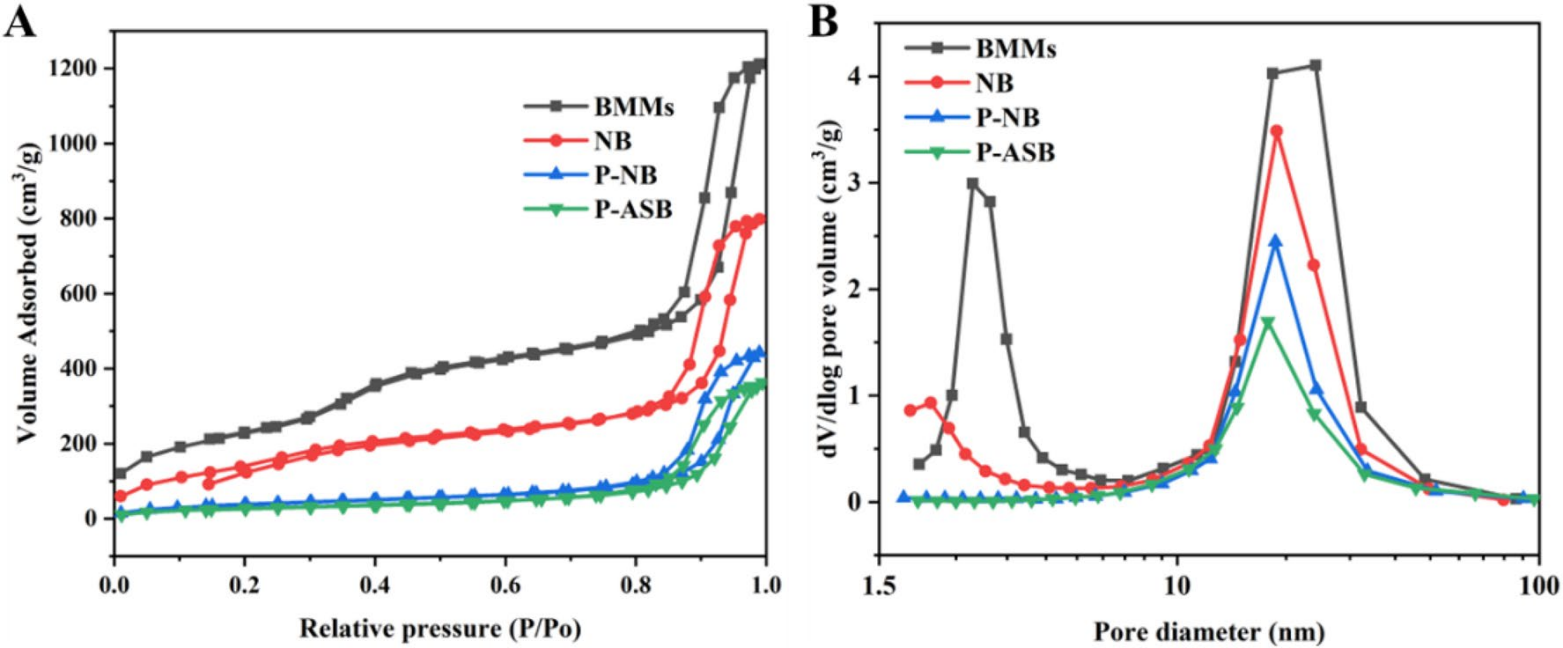
N_2_ adsorption–desorption isotherms (**A**) and pore size distribution (**B**) of P-ASB NPs and control samples.

The porosity characterizations of P-ASB NPs were studied by the nitrogen (N_2_) adsorption–desorption technique. Figure 4 show that the BMMs and NB NPs exhibited a typical Langmuir IV isotherm containing two hysteresis loops, thus implying that they contained a double-channel mesoporous structure. After loading Pro and grafting disulfide bonds and AC, the hysteresis loop at 0.3 < *P*/*P*_0_ < 0.5 disappeared, indicating that the mesoporous channels were occupied by Pro. The specific surface area and pore volume of NPs decreased significantly from 566.10 m^2^/g and 1.18 cm^3^/g (NB NPs) to 132.94 m^2^/g and 0.56 cm^3^/g (P-ASB NPs), respectively (Table 1). Figure 4B shows that the small mesoporous channel peak of P-ASB NPs disappeared and the strength of its large mesoporous channel decreased. These results further indicated that the channels were occupied by Pro and were well covered with the disulfide cross-linked aminocellulose coating. The 3D size of the prochloraz molecule is 1.03 nm in length and 0.98 nm in width. It can be fully adsorbed and loaded by the pores of BMMs.

**Figure 5:**
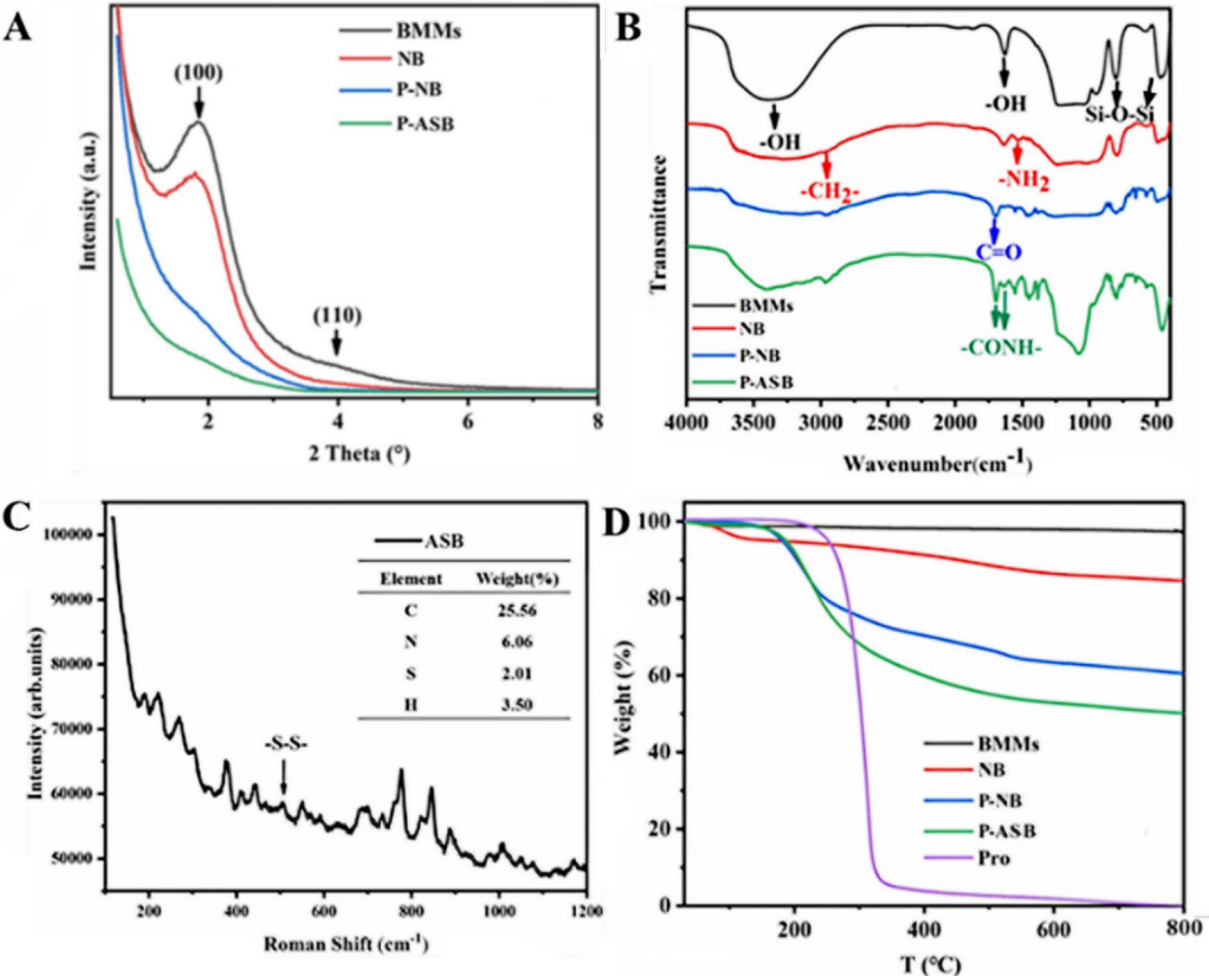
Small-angle XRPD patterns (**A**), FTIR spectra (**B**), Raman spectrum and element contents (**C**) and TGA profiles (**D**) of P-ASB NPs and control samples.

We performed XRPD analysis to investigate the crystal structure of NPs (Fig. 5A).

The (100) diffraction peak of BMMs at 2θ = 1.84° was the classic peak of BMMs, suggesting that nanoparticles had a highly organized mesoporous structure. After modification by APTES, the diffraction peak intensity of NB at (100) decreased; thus indicating that the introduction of organic functional groups affected the pore structure of BMMs. After Pro loading, the (100) diffraction peak intensity of P-NB dropped sharply, which implied that Pro molecules were successfully storaged into the channels of BMMs. Covalently coupled with disulfide bonds and AC, the (100) diffraction peak of P-ASB completely disappeared, which indicating that the introduction of gatekeepers led to a decrease in the order of its structure.

To further explore the assembly process of P-ASB NPs, the nanocomposite was characterized by a Nexus 470 FTIR spectrometer. Figure 5B show that all nanoparticles had an absorption peak of Si–O–Si stretching vibration at 466 cm^−1^ and 798 cm^−1^. After APTES modification, the NB showed a characteristic bending vibration peak of –NH_2_ at 1534 cm^−1^. The amide bond stretching vibration peak of Pro at 1698 cm^−1^ can also be found in the spectrum of P-NB. These data confirm that Pro was successfully entrapped in the nanoparticles. The characteristic peaks of amide bonds (–CONH–) at 1640 cm^−1^ and that of methylene (–CH_2_–) at 2990 cm^−1^ were enhanced in the spectrum of P-ASB, indicating that the carboxyl group of 2,2′-disulfodiacetic acid was well connected to the amino group of P-NB and the amino group of AC. In addition, as shown in Fig. 5C, Raman analysis and organic element analysis of P-ASB NPs showed that the disulfide bond was successfully introduced into the framework of P-ASB. Finally, the loading ratio of P-ASB NPs was determined with TG analysis (Fig. 5D). The weight loss of NPs in the range of 30–150 °C was blamed to the evaporation of water, while that after 150 °C was attributed to the decomposition of the organic groups on the surface of NPs. BMMs maintained a constant weight and possessed high thermal stability from 150 to 800 °C. The total weight loss rates of NB, P-NB, and P-ASB were 10.59%, 38.14% and 50.01%, respectively. The weight losses of the nanoparticles gradually increased, indicating successful loading of Pro and the modification of the BMMs by the gatekeeper material. Therefore, the loading rate of P-ASB NPs was 27.6%, which is almost consistent with HPLC data (28.5%).

### Release behavior

**Figure 6:**
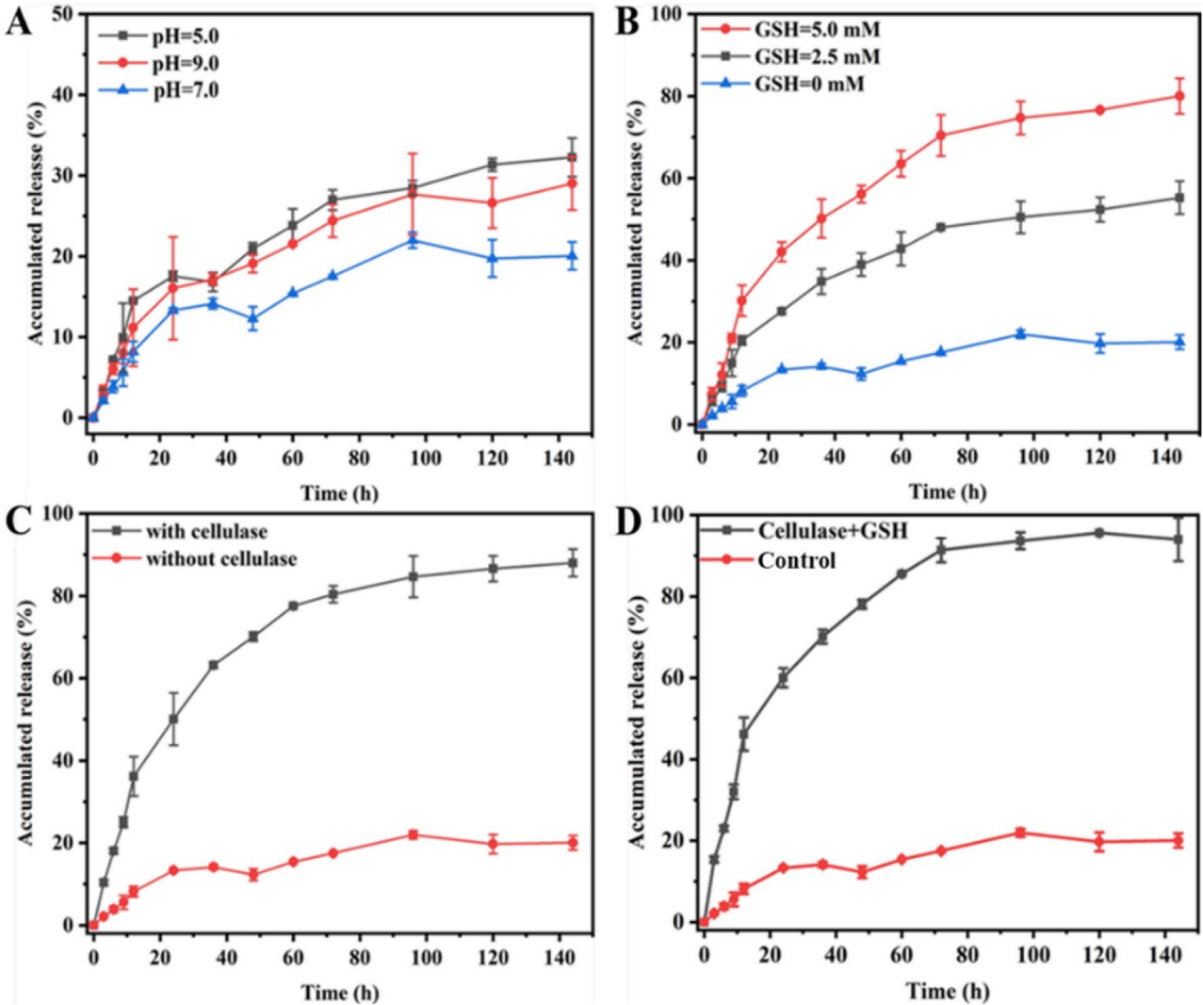
Effects of pH value (**A**), GSH concentrations (**B**), cellulase (**C**) and cellulase + GSH (D) on the release behaviors of Pro from P-ASB NPs.

The release behaviors of P-ASB NPs were first explored at various pH values. The cumulative Pro release behaviors from P-ASB NPs in release media with pH 5.0, 7.0, and 9.0 are shown in Fig. 6A. The cumulative release rates of Pro were all below 32.0% after 144 h, which may be due to the stability of coatings under acid–base conditions as well as the good encapsulation performance of the pore-sealing material.

We next explored the effects of different GSH concentrations and cellulase on the release behaviors of P-ASB NPs. The release rate of Pro from NPs gradually improved with increasing GSH concentration (Fig. 6B). The release rate of Pro was 80.0% at 144 h when the GSH concentration was 5.0 mM. This redox release characteristic of NPs was mainly due to the disulfide bond (–S–S–) integrated in P-ASB NPs, which acts as a bridge switch, thus breaking the sulfhydryl group (–SH–) in GSH. Cellulase is a major class of polysaccharide hydrolase that is secreted during the colonization and development of pathogens. Figure 6C clearly shows the effect of cellulase on the Pro release from P-ASB NPs. In the presence of cellulase, the cumulative release ratios of Pro increased from 50.2% at 24 h to 90.9% at 144 h. Cellulase degraded the aminocellulose on the surface of the nanocarrier and significantly accelerated the release of Pro. Finally, the effect of the coexistence of cellulase and GSH on the release behavior of the nano pesticide was explored (Fig. 6D). The initial release of Pro molecules was the fastest when the two stimulus factors co-existed (5.0 mM GSH and cellulase). The release of Pro reached 60.1% after 24 h and 94.0% after 144 h, while that was only 18.9% in distilled water at 144 h.

To probe into the release mechanism of P-ASB NPs deeply, the release kinetics parameters of NPs were delved with Zero-order, First-order, Higuchi, and Ritger–Peppas models^10,34^. As demonstrated in Table 2, the First-order model gave the most rational interpretation of the Pro release mechanism, basing on the correlation coefficient (R^2^) obtained. The release parameter of Pro was 0.045 in the presence of cellulase and GSH, while it was only 0.033 in distilled water, indicating that Pro was released relatively faster in the presence of cellulase and GSH. The structure of the nanoparticles was destroyed due to the interaction of GSH and cellulase on disulfide bonds (–S–S–) and aminocellulose, respectively. These results indicated that P-ASB NPs possessed the redox and cellulase dual-responsive characteristics and can effectively respond to the changes of pathogenic microenvironment and intelligently release pesticide molecules.

**Table 2:** Release kinetics parameters of P-ASB NPs in the present of GSH and cellulase.

Group	Fitting models	Kinetic Equations	R^2^
GSH + cellulase	Zero-order	Q = 0.61t + 31.00	0.71
	First-order	Q = 93.47 (1 − e^−0.045t^)	0.99
	Higuchi	Q = 8.60t1/2 + 9.16	0.91
	Ritger–Peppas	Q = 17.26t^0.37^	0.94
Blank control	Zero-order	Q = 0.14t + 5.27	0.77
	First-order	Q = 19.96 (1 − e^−0.033t^)	0.94
	Higuchi	Q = 1.87t1/2 + 0.72	0.92
	Ritger–Peppas	Q = 2.68t^0.43^	0.93

### Photostability

**Figure 7:**
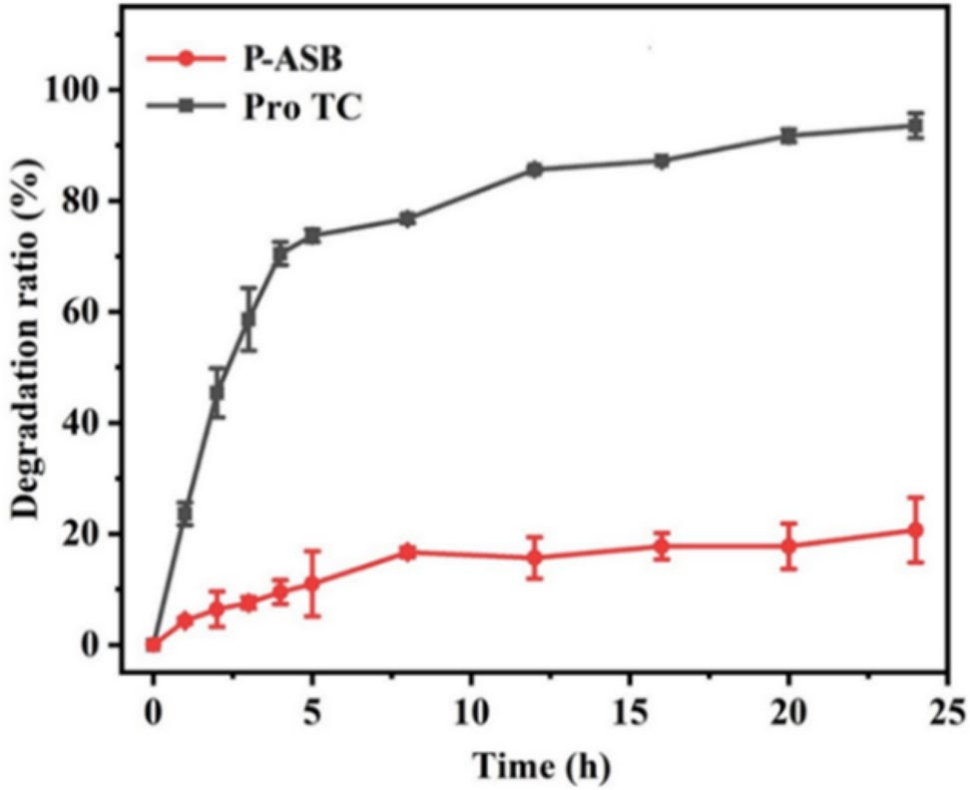
Photostability of P-ASB NPs under UV-light irradiation.

Pro TC is easily degraded under UV light, which results in a significant reduction in effective utilization^35^. One of the best ways to avoid this problem is to encapsulate Pro with nanocarriers. Figure 7 shows that the photodegradation rate of Pro TC was higher than 94.1% after 24 h of UV-light irradiation, while the photodegradation rate of Pro in the ASB nanocarrier was only about 20.0%. The results indicated that the nanocarrier has a good shielding effect on UV light and can effectively reduce the photodegradation of pesticide molecules in its pores. The shielding properties of nanocarriers against UV light may be because they can absorb or reflect most of the UV light, thus greatly reducing the exposure of Pro to UV light^36^.

### Foliage wettability

**Figure 8:**
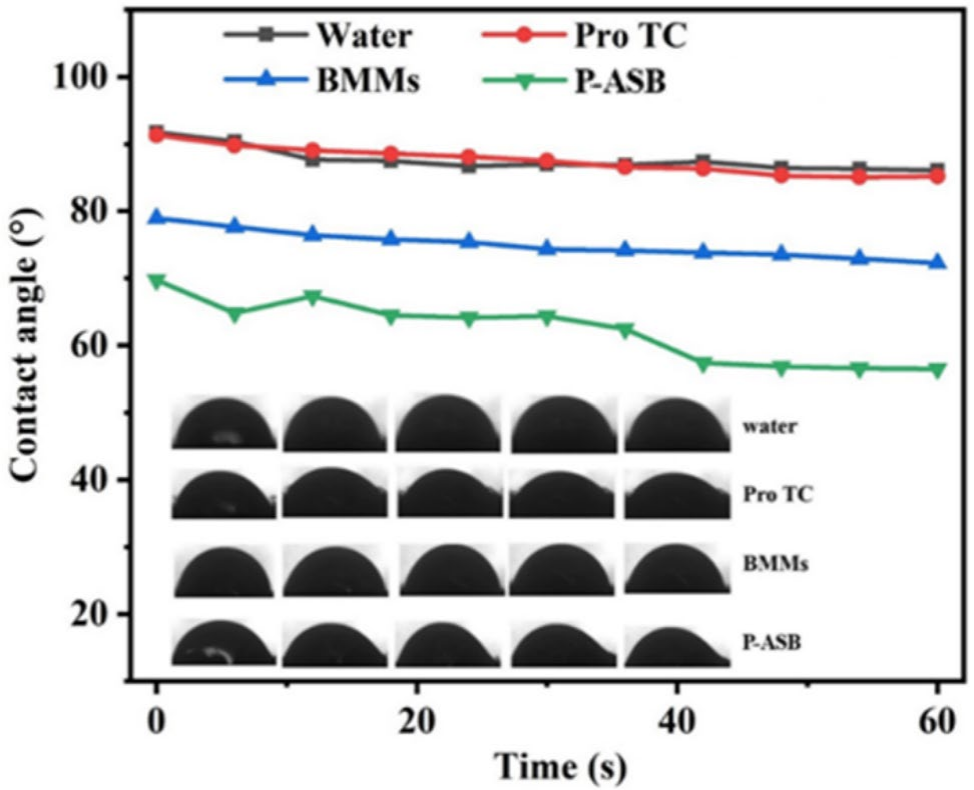
Contact angle images and values of P-ASB NPs and control samples on cucumber leaves.

One important reason for the low utilization rate of pesticides is that the pesticide liquid easily rolls off the leaf surface. The deposition and utilization efficiency of pesticides can be improved by using the interface adhesion property of nanoparticles on crop leaves^37,38^. Figure 8 shows that the contact angles of Pro were relatively unchanged, at 92°. After AC coating, the contact angle of P-ASB decreased drastically with time and was only 67° after 1 min. This may be because of the size effect of NPs and the electrostatic or hydrogen bonding interactions between amino groups and polar groups on the leaves. In addition, topological structure formation between the wax layer and the P-ASB NPs may also enhance nanoparticle adhesion ability^39^. This indicates that P-ASB NPs has good affinity on crop leaves and can make the Pro spread better on the leaf surface and thus reduce the possibility of water droplets falling off the leaf surface, which is consistent with the previous report^40^. This in turn improves the deposition and utilization efficiency of Pro.

### Uptake of NPs in fungi

**Figure 9:**
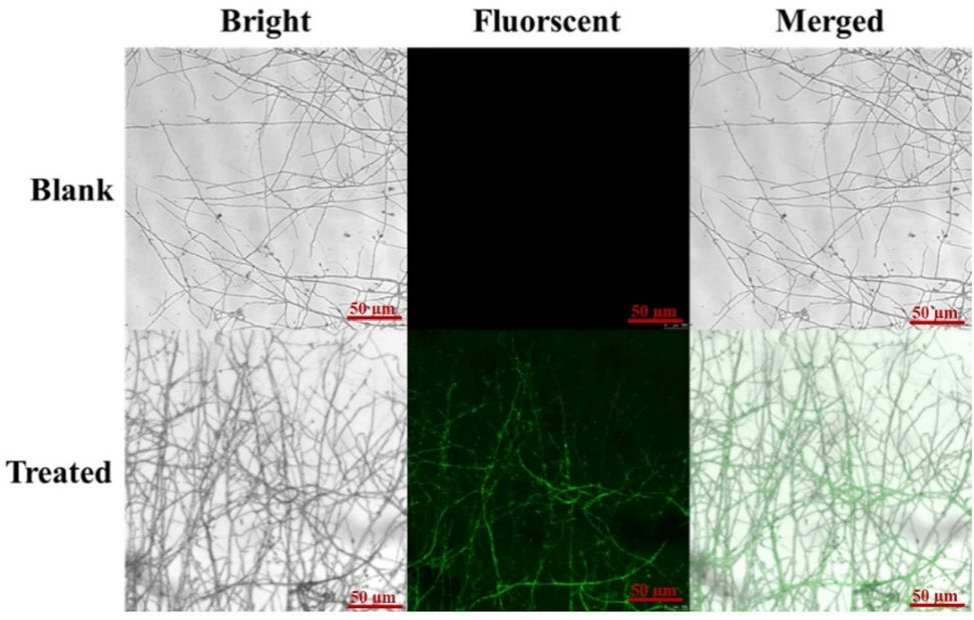
Fluorescence images of *R*. *solani* incubated with ASB/FITC and blank control for 7 days. The laser excitation wavelength was 488 nm.

The nanocarrier can be well absorbed and transferred by fungi, which is important to improve the biological activity of encapsulated pesticides, especially for non-systemic pesticides such as prochloraz^10^. To observe the absorption and transfer of ASB by fungal mycelia more intuitively, ASB was fluorescently labeled with FITC to make it visible in mycelia. *R*. *solani* growing on PDA were then treated with ASB/FITC aqueous suspension. After 7 days of incubation, the mycelia-covered slides were carefully removed for imaging observation with CLSM (Fig. 9). The blank mycelium had no fluorescence signal, but the mycelium treated with ASB/FITC showed a clear fluorescence signal under CLSM, thus indicating that ASB can be used as an ideal carrier to transport fungicides to fungi and improve their bioavailability.

### Fungicidal activity

**Figure 10:**
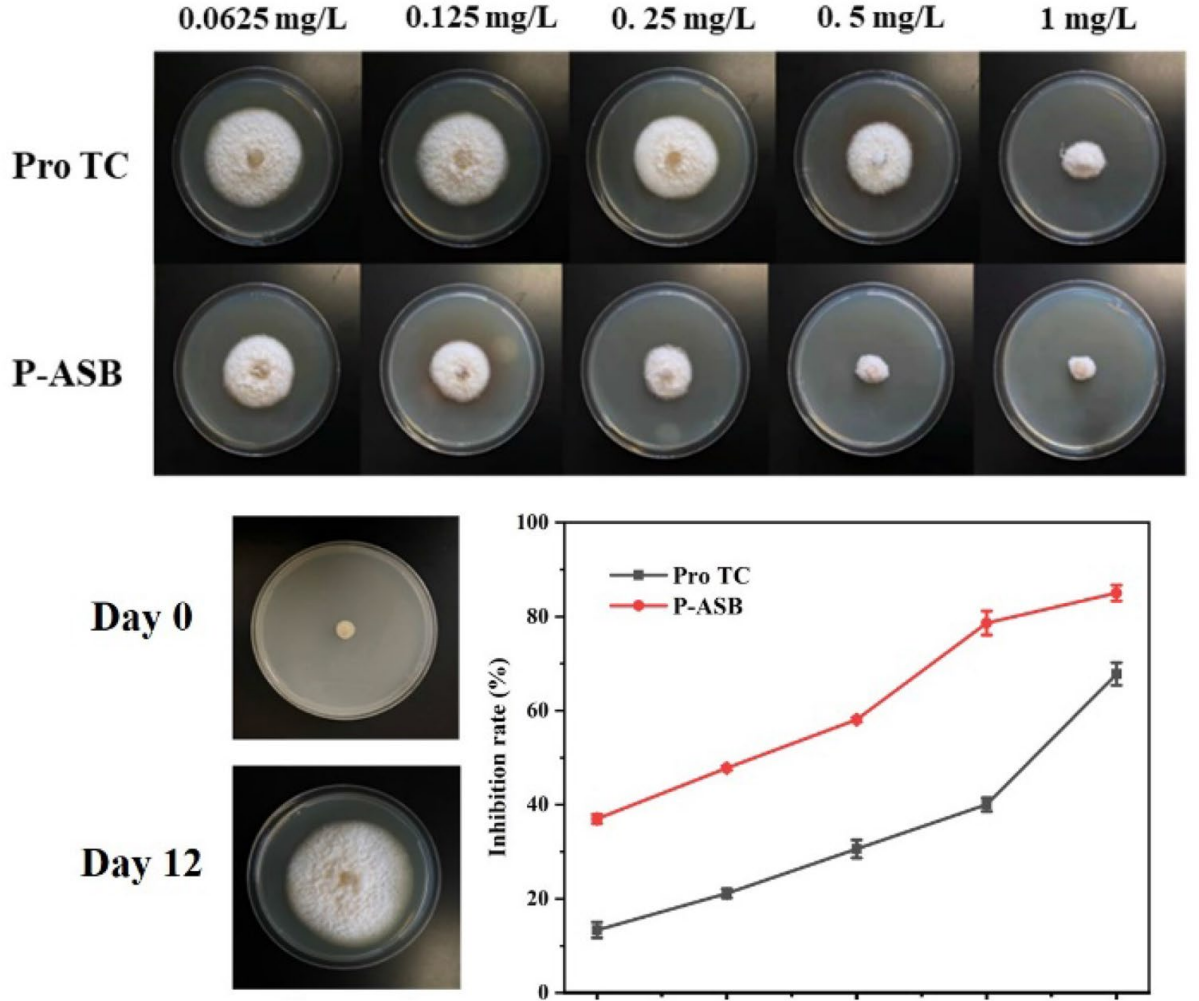
Fungicidal activity and inhibitory rates of P-ASB NPs in vitro against *R. solani* on the 12th day.

The bioactivity of P-ASB NPs against *R. solani* was determined via the growth-rate method. The growth of *R. solani* in different Pro-as-an-active-ingredient concentrations is shown in Fig. 10. The fungicidal activity of P-ASB NPs against *R. solani* was concentration-dependent within 12 days and was better than that of Pro TC. The inhibition rate of P-ASB NPs (78.6%) was higher than that of Pro TC (40.0%) when the effective concentration of Pro was 0.5 mg/L. Accordingly, the IC50 values of P-ASB NPs (0.13 mg/L) were lower than those of Pro TC (0.58 mg/L) (Table 3). This is because the disulfide bond and AC grafted on P-ASB NPs could be degraded by GSH. In addition, the cellulase secreted by *R. solani* led to a sustained and on-demand release of Pro from the nanocarriers. This redox and cellulase dual-response nano-delivery significantly inhibited the growth of *R. solani* and improved the effective utilization of the pesticide.

**Table 3:** Fungicidal activity of P-ASB NPs against *R. solani* at 12 days.

Fungicides	Toxicity regression equation	IC_50_ (mg/L)	95% Confidence intervals	Relative virulence
P-ASB	y = 0.42x + 0.87	0.13	0.10–0.19	1.25
Pro TC	y = 0.43x + 0.60	0.58	0.37–0.90	1.00

### Biosafety evaluation

**Figure 11:**
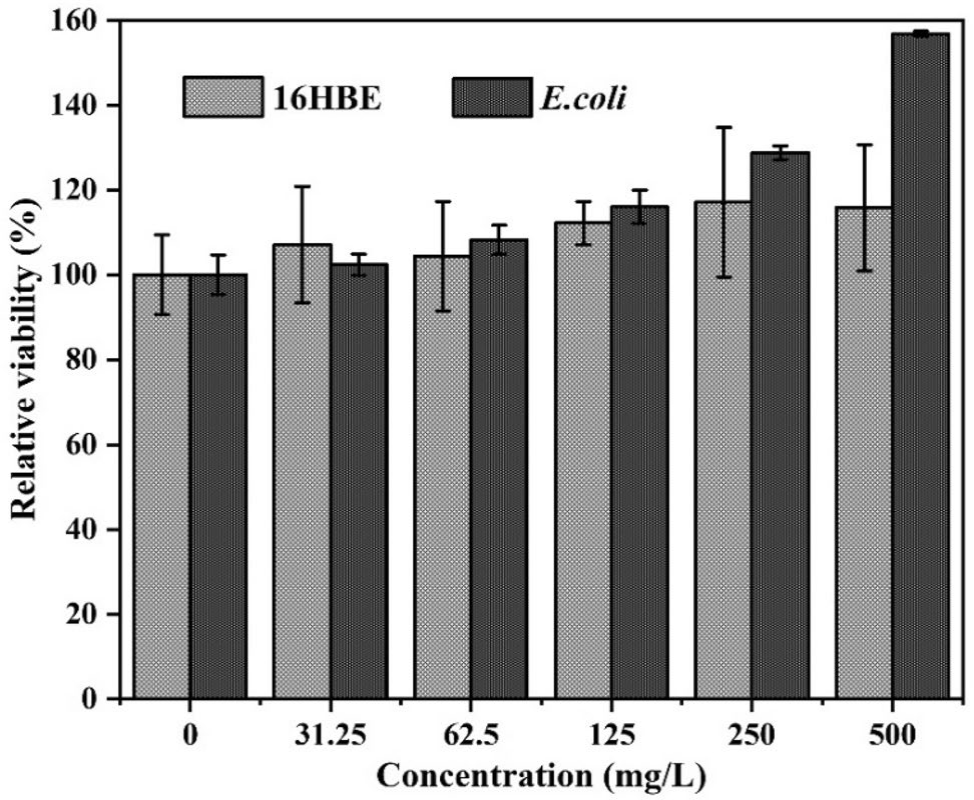
Biosafety evaluations of different concentrations of ASB nanocarriers.

Biosafety is an essential factor for the large-scale application of nano-pesticides in the future^7^. We evaluated the biosafety of ASB nano vectors by toxicological methods. Figure 11 shows that ASB did not inhibit the growth of 16HBE cells and *E. coli* in the concentration range of 0–500 mg/L. The relative viabilities of the cells and *E. coli* were 115.78% and 156.84%, respectively, when 500 mg/L of ASB NPs were added in the media, which promoted the growth of *E. coli* and 16HBE cells due to the introduction of amicellulose polysaccharides. These data indicated that this dual-responsive nanocarrier has ideal biocompatibility and high biosafety.

## Conclusion

Mesoporous silica and aminocellulose are cost-effective and have good biosafety. Their combination resulted in controlled-release nano-pesticides with good applicability and research value^41,42^. Herein, a P-ASB nano-delivery system with redox/cellulase dual response was fabricated to control the infection caused by *R. solani*. Pro was encapsulated into the pores of BMMs nanocarrier coated with the 2,2′-dithiodiacetic acid smart bridge and “SS-AC” gatekeeper. The P-ASB NPs had a uniformly spherical morphology and a high Pro-loading rate of 28.5%. The release of Pro in P-ASB NPs was positively correlated with the concentrations of GSH and cellulose. In release media with GSH, the reductive cleavage of the disulfide bond resulted in controlled/sustained release of Pro. Meanwhile, the aminocellulose coating on the surface of the nanocarrier was degraded by cellulose in fungi, which also accelerated the release of Pro. This functional nano-pesticide has excellent water dispersibility, photostability, and foliar adhesion properties. The uptake and conduction of nanocomposites in vivo by *R. solani* improved the efficient utilization of Pro as a non-systemic pesticide. The antifungal activity showed that P-ASB NPs possessed excellent characteristics to prevent fungal disease compared to Pro TC. Biosafety experiments with the nanocarriers showed that they can reduce the pollution of conventional pesticides and environmental risk. Mesoporous silica has been widely used in various fields due to its low production cost and favorable biosafety. Multifunctional nanopesticides can be expected to produce on a large scale, and bring good economic benefits in agricultural^43^. In conclusion, this redox/cellulase dual-responsive nano-delivery system provides ideas for the design and preparation of new formulations of pesticides with high efficiency and promotes the development of green and sustainable agriculture.

## Data Availability

The data generated during the current study are available from the corresponding author on reasonable request.
